# Crystal structure of new organically templated copper sulfate with 2-amino­pyridinium

**DOI:** 10.1107/S2056989015018629

**Published:** 2015-10-10

**Authors:** Tamara J. Lukianova, Vasyl Kinzhybalo, Adam Pietraszko

**Affiliations:** a Institute of Low Temperature and Structure Research, Polish Academy of Sciences, Okolna str. 2, PO Box 1410, 50-950 Wroclaw, Poland

**Keywords:** crystal structure, organically templated materials, 2-amino­pyridine, sulfates, hydrogen bonding, π–π inter­actions

## Abstract

The title compound, (C_5_H_7_N_2_)_2_[Cu(H_2_O)_6_](SO_4_)_2_·4H_2_O [systematic name: bis(2-aminopyridinium) hexaaquacopper(II) bis(sulfate) tetrahydrate], comprises axially elongated hexa­aqua-coordinated octa­hedral Cu^II^ ions located on an inversion centre, non-coordinating sulfate anions, 2-amino­pyridinium cations and lattice water mol­ecules. The crystal structure is built of successive inorganic and organic layers extending parallel to (001) that are connected by an extensive three-dimensional hydrogen-bonded network of the type O—H⋯O and N—H⋯O, as well as π–π inter­actions [centroid–centroid distance 3.4140 (14) Å, offset 0.277 Å].

## Related literature   

For applications of 2-amino­pyridine, see: Windholz (1976 ▸). For 2-amino­pyridinium sulfate, see: Jebas *et al.* (2006 ▸). For other compounds with copper(II), see: Naïli *et al.* (2006 ▸); Rekik *et al.* (2006 ▸).
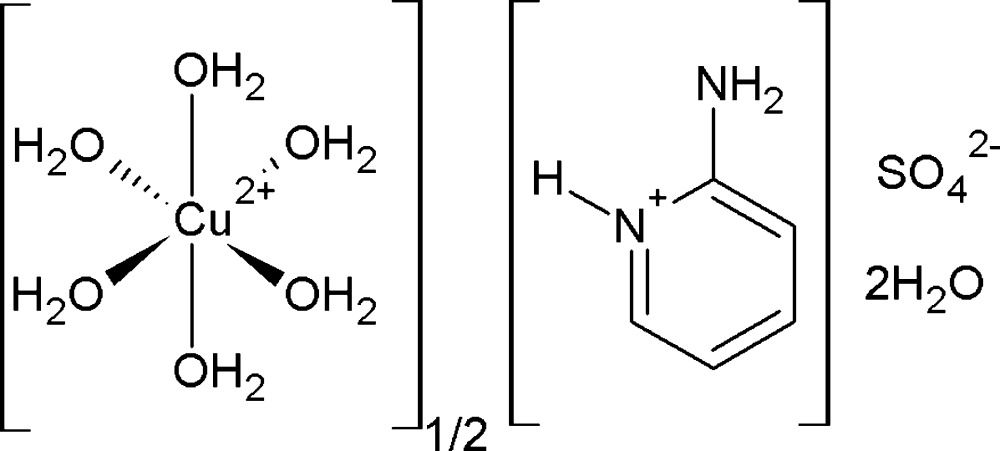


## Experimental   

### Crystal data  

(C_5_H_7_N_2_)_2_[Cu(H_2_O)_6_](SO_4_)_2_·4H_2_O
*M* *_r_* = 626.07
Triclinic, 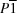

*a* = 7.115 (3) Å
*b* = 8.211 (3) Å
*c* = 12.561 (4) Å
α = 91.83 (3)°
β = 104.59 (3)°
γ = 114.57 (3)°
*V* = 638.0 (4) Å^3^
*Z* = 1
Mo *K*α radiation
μ = 1.10 mm^−1^
*T* = 295 K
0.35 × 0.14 × 0.13 mm

### Data collection  

Rigaku Oxford Diffraction Xcalibur, Sapphire2 diffractometer
Absorption correction: multi-scan (*CrysAlis PRO*; Rigaku Oxford Diffraction, 2015 ▸) *T*_min_ = 0.720, *T*_max_ = 1.000
7926 measured reflections
3173 independent reflections
2268 reflections with *I* > 2σ(*I*)
*R*_int_ = 0.038

### Refinement  

*R*[*F* ^2^ > 2σ(*F* ^2^)] = 0.044
*wR*(*F* ^2^) = 0.094
*S* = 1.03
3173 reflections
160 parameters
H-atom parameters constrained
Δρ_max_ = 0.36 e Å^−3^
Δρ_min_ = −0.41 e Å^−3^

### 

Data collection: *CrysAlis PRO* (Rigaku Oxford Diffraction, 2015 ▸); cell refinement: *CrysAlis PRO*; data reduction: *CrysAlis PRO*; program(s) used to solve structure: *SHELXS2014/7* (Sheldrick, 2008 ▸); program(s) used to refine structure: *SHELXL2014/7* (Sheldrick, 2015 ▸); molecular graphics: *DIAMOND* (Brandenburg *et al.*, 1997 ▸); software used to prepare material for publication: *OLEX2* (Dolomanov *et al.*, 2009 ▸).

## Supplementary Material

Crystal structure: contains datablock(s) I. DOI: 10.1107/S2056989015018629/hp2072sup1.cif

**Fig. 1.**
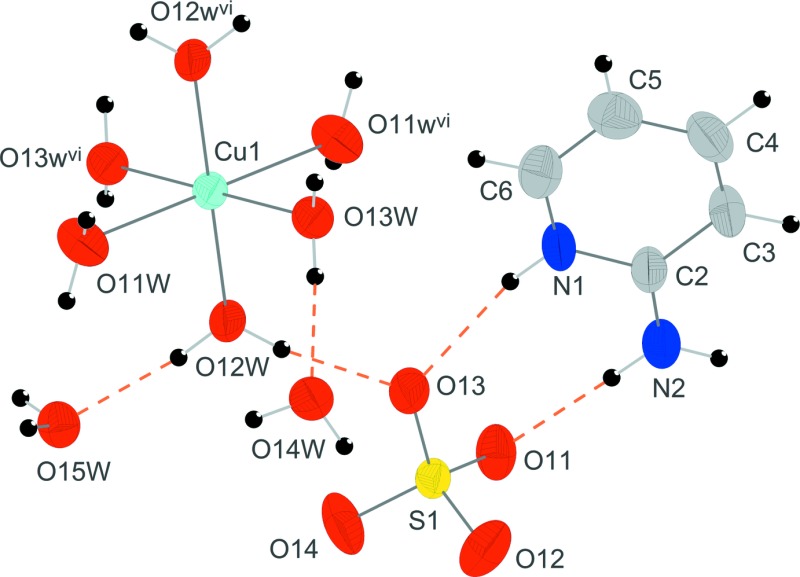
The asymmetric unit of the title compound, showing the crystallographic numbering scheme. Displacement ellipsoids are drawn at the 50% probability level. Hydrogen bonds are denoted by orange dashed lines. [Symmetry codes: (vi) -x + 1, -y + 1, -z + 1].

Structure factors: contains datablock(s) I. DOI: 10.1107/S2056989015018629/hp2072Isup2.hkl

vi -x 1 -y 1 -z 1 . DOI: 10.1107/S2056989015018629/hp2072fig1.tif
The asymmetric unit of the title compound, showing the crystallographic numbering scheme. Displacement ellipsoids are drawn at the 50% probability level. Hydrogen bonds are denoted by orange dashed lines. [Symmetry codes: (*vi*) *-x* + *1*, *-y* + *1*, *-z* + *1*].

**Fig. 2.**
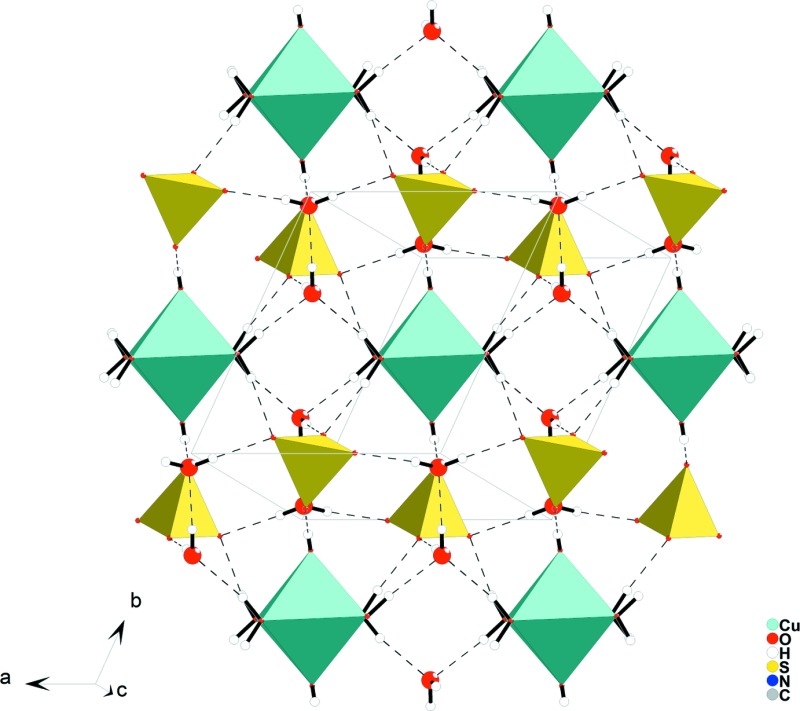
View of inorganic layers along perpendicular to this layer direction (c*). Dashed lines indicate the hydrogen bonds.

. DOI: 10.1107/S2056989015018629/hp2072fig2.tif
View of inorganic layers along perpendicular to this layer direction (a*). Dashed lines indicate the hydrogen bonds.

**Fig. 3.**
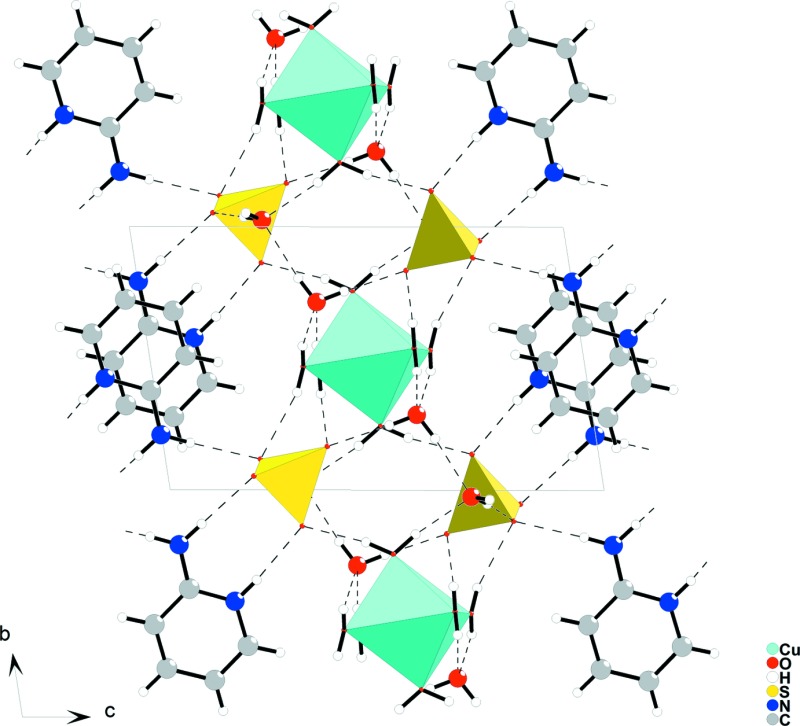
The molecular arrangement in (C5H7N2)2[CuII(H2O)6](SO4)2·4H2O viewed along [100]. Dashed lines represent hydrogen bonds.

5 7 2 2 II 2 6 4 2 2 . DOI: 10.1107/S2056989015018629/hp2072fig3.tif
The mol­ecular arrangement in (C_5_H_7_N_2_)_2_[Cu^II^(H_2_O)_6_](SO_4_)_2_·4H_2_O viewed along [100]. Dashed lines represent hydrogen bonds.

CCDC reference: 1429506

Additional supporting information:  crystallographic information; 3D view; checkCIF report

## Figures and Tables

**Table 1:** Hydrogen-bond geometry (, )

*D*H*A*	*D*H	H*A*	*D* *A*	*D*H*A*
O11*W*H11*A*O14*W* ^i^	0.84	2.00	2.821(3)	167
O11*W*H11*B*O12^ii^	0.84	2.22	3.032(4)	162
O12*W*H12*A*O15*W*	0.84	1.88	2.719(3)	172
O12*W*H12*B*O13	0.84	1.85	2.677(3)	171
O13*W*H13*A*O14^iii^	0.84	1.90	2.733(3)	174
O13*W*H13*B*O14*W*	0.84	1.88	2.706(3)	168
N1H1O13	0.86	2.03	2.855(3)	160
N2H2*A*O11	0.86	2.01	2.869(3)	176
N2H2*B*O12^iv^	0.86	2.05	2.914(3)	178
O14*W*H14*A*O15*W* ^ii^	0.84	1.92	2.758(3)	174
O14*W*H14*B*O14^ii^	0.84	1.90	2.738(3)	176
O15*W*H15*A*O11^v^	0.84	1.93	2.761(3)	169
O15*W*H15*B*O12^ii^	0.84	1.93	2.760(3)	170

Symmetry codes: (i) 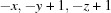; (ii) 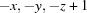; (iii) 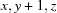; (iv) 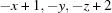; (v) 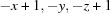.

## supplementary crystallographic information

### S1. Comment

Crystal structure of I is composed of 2-amino­pyridinium (2ap) cations, isolated sulfate anions, metal cations o­cta­hedrally coordinated by six water molecules [Cu(H_2_O)_6_]^2+^ and uncoordinated water molecules. The atom labeling scheme for compound I is shown in Fig. 1. The asymmetric unit contains one half of Cu atom (lies on a center of inversion) along with three water molecules coordinated to it, one sulfate group, one protonated amine and two solvation water molecules. The Cu ion environment shows considerable axial deformation to tetra­gonal bipyramidal due to Jahn-Teller effect. The Cu–O12W and Cu–O13W distances are equal to 1.935 (2) and 1.9790 (18) Å, respectively, and the Cu–O11W distance is strongly elongated to 2.398 (2) Å. The distances within the [Cu(H_2_O)_6_]^2+^ o­cta­hedron are comparable to those observed in other compounds (Naïli *et al.*, 2006; Rekik *et al.*, 2006). The crystal packing consists of successive organic and inorganic layers parallel to 0*xy* plane. Inorganic layers are stabilized by a series of O–H···O hydrogen bonds (Table 1 and Fig. 2). Organic layers are built of π-π inter­acting stacks of 2ap cations (*Cg*···*Cg* 3.4140 (14) Å, offset 0.277 Å) connected to inorganic layers through N–H···O hydrogen bonds (Table 1 and Fig. 3).

### S2. Experimental

The title compound was synthesized by the following method. 2-amino­pyridine (0.19 g, 2 mmol) was dissolved in 4 ml double distilled water to obtain solution A. The pH of the solution was adjusted to 2.5, by the addition of 30% sulfuric acid. Copper sulfate (0.149 g, 6 mmol) was dissolved in 3 ml double distilled water to obtain solution B. Solution A was added on solution B. The resulting solution was kept at room temperature. The green crystals of the title compound were obtained by slow evaporation during the period of several months.

### S3. Refinement

The H atoms of water molecules were located from difference Fourier maps and were refined with O–H distances restrained to 0.840 (2) Å and *U*_iso_(H) = 1.5 *U*_eq_(O). In final refinement cycles H atoms of water were let to ride on parent O atom (AFIX 3).

### Crystal data

**Table:**

(C_5_H_7_N_2_)_2_[Cu(H_2_O)_6_](SO_4_)_2_·4H_2_O	*Z* = 1
*M**_r_* = 626.07	*F*(000) = 327
Triclinic, *P*1	*D*_x_ = 1.629 Mg m^−^^3^
*a* = 7.115 (3) Å	Mo *K*α radiation, λ = 0.71073 Å
*b* = 8.211 (3) Å	Cell parameters from 2602 reflections
*c* = 12.561 (4) Å	θ = 3.3–27.4°
α = 91.83 (3)°	µ = 1.10 mm^−^^1^
β = 104.59 (3)°	*T* = 295 K
γ = 114.57 (3)°	Block, green
*V* = 638.0 (4) Å^3^	0.35 × 0.14 × 0.13 mm

### Data collection

**Table:**

Rigaku Oxford Diffraction Xcalibur, Sapphire2 diffractometer	3173 independent reflections
Radiation source: fine-focus sealed X-ray tube, Enhance (Mo) X-ray Source	2268 reflections with *I* > 2σ(*I*)
Graphite monochromator	*R*_int_ = 0.038
Detector resolution: 8.2214 pixels mm^-1^	θ_max_ = 29.4°, θ_min_ = 3.0°
ω scans	*h* = −9→6
Absorption correction: multi-scan (*CrysAlis PRO*; Rigaku Oxford Diffraction, 2015)	*k* = −10→11
*T*_min_ = 0.720, *T*_max_ = 1.000	*l* = −15→17
7926 measured reflections	

### Refinement

**Table:**

Refinement on *F*^2^	0 restraints
Least-squares matrix: full	Hydrogen site location: mixed
*R*[*F*^2^ > 2σ(*F*^2^)] = 0.044	H-atom parameters constrained
*wR*(*F*^2^) = 0.094	*w* = 1/[σ^2^(*F*_o_^2^) + (0.0324*P*)^2^ + 0.2243*P*] where *P* = (*F*_o_^2^ + 2*F*_c_^2^)/3
*S* = 1.03	(Δ/σ)_max_ < 0.001
3173 reflections	Δρ_max_ = 0.36 e Å^−^^3^
160 parameters	Δρ_min_ = −0.41 e Å^−^^3^

### Special details

**Table:**

Geometry. All e.s.d.'s (except the e.s.d. in the dihedral angle between two l.s. planes) are estimated using the full covariance matrix. The cell e.s.d.'s are taken into account individually in the estimation of e.s.d.'s in distances, angles and torsion angles; correlations between e.s.d.'s in cell parameters are only used when they are defined by crystal symmetry. An approximate (isotropic) treatment of cell e.s.d.'s is used for estimating e.s.d.'s involving l.s. planes.

### Fractional atomic coordinates and isotropic or equivalent isotropic displacement parameters (Å^2^)

**Table:**

	*x*	*y*	*z*	*U*_iso_*/*U*_eq_	
Cu1	0.5000	0.5000	0.5000	0.03035 (15)	
O11W	0.1945 (3)	0.4686 (3)	0.35088 (16)	0.0476 (5)	
H11A	0.1489	0.5468	0.3566	0.071*	
H11B	0.0825	0.3704	0.3272	0.071*	
O12W	0.3772 (3)	0.2434 (2)	0.50851 (15)	0.0482 (6)	
H12A	0.3051	0.1679	0.4500	0.072*	
H12B	0.3718	0.1983	0.5673	0.072*	
O13W	0.3680 (3)	0.5392 (2)	0.61353 (14)	0.0359 (4)	
H13A	0.3521	0.6347	0.6187	0.054*	
H13B	0.2510	0.4506	0.6110	0.054*	
S1	0.34958 (11)	−0.04901 (9)	0.72795 (5)	0.03015 (17)	
O11	0.5437 (3)	−0.0526 (3)	0.80214 (15)	0.0434 (5)	
O12	0.1755 (3)	−0.1167 (3)	0.78078 (18)	0.0541 (6)	
O13	0.3945 (4)	0.1366 (3)	0.70846 (16)	0.0493 (6)	
O14	0.2833 (4)	−0.1658 (3)	0.62203 (16)	0.0566 (6)	
N1	0.6656 (4)	0.4231 (3)	0.89059 (17)	0.0385 (6)	
H1	0.6136	0.3421	0.8326	0.046*	
N2	0.6983 (4)	0.2085 (3)	0.99866 (19)	0.0462 (6)	
H2A	0.6458	0.1297	0.9395	0.055*	
H2B	0.7349	0.1784	1.0629	0.055*	
C2	0.7227 (4)	0.3756 (4)	0.9910 (2)	0.0364 (6)	
C3	0.8066 (5)	0.5115 (4)	1.0846 (2)	0.0484 (8)	
H3	0.8482	0.4852	1.1557	0.058*	
C4	0.8263 (5)	0.6786 (4)	1.0711 (3)	0.0527 (8)	
H4	0.8812	0.7670	1.1334	0.063*	
C5	0.7659 (5)	0.7227 (4)	0.9654 (3)	0.0510 (8)	
H5	0.7806	0.8391	0.9566	0.061*	
C6	0.6855 (5)	0.5915 (4)	0.8760 (3)	0.0477 (8)	
H6	0.6441	0.6171	0.8046	0.057*	
O14W	−0.0364 (3)	0.2860 (3)	0.59552 (16)	0.0432 (5)	
H14A	−0.0658	0.1958	0.6291	0.065*	
H14B	−0.1086	0.2458	0.5285	0.065*	
O15W	0.1567 (3)	0.0248 (2)	0.30825 (15)	0.0381 (5)	
H15A	0.2371	0.0360	0.2675	0.057*	
H15B	0.0585	0.0513	0.2734	0.057*	

### Atomic displacement parameters (Å^2^)

**Table:**

	*U*^11^	*U*^22^	*U*^33^	*U*^12^	*U*^13^	*U*^23^
Cu1	0.0418 (3)	0.0233 (2)	0.0246 (2)	0.0126 (2)	0.0103 (2)	0.00304 (17)
O11W	0.0435 (13)	0.0422 (12)	0.0502 (13)	0.0187 (10)	0.0034 (10)	−0.0031 (9)
O12W	0.0811 (16)	0.0242 (10)	0.0267 (10)	0.0126 (10)	0.0132 (10)	0.0046 (8)
O13W	0.0416 (11)	0.0323 (10)	0.0345 (10)	0.0166 (9)	0.0121 (9)	0.0024 (8)
S1	0.0370 (4)	0.0289 (4)	0.0247 (3)	0.0156 (3)	0.0071 (3)	0.0055 (3)
O11	0.0407 (12)	0.0640 (14)	0.0317 (10)	0.0304 (11)	0.0077 (9)	0.0081 (9)
O12	0.0433 (13)	0.0771 (16)	0.0562 (14)	0.0330 (12)	0.0231 (11)	0.0360 (12)
O13	0.0755 (16)	0.0315 (11)	0.0321 (11)	0.0188 (11)	0.0090 (10)	0.0076 (8)
O14	0.0838 (17)	0.0504 (13)	0.0325 (11)	0.0404 (13)	−0.0057 (11)	−0.0096 (9)
N1	0.0453 (15)	0.0451 (15)	0.0225 (11)	0.0202 (12)	0.0053 (10)	0.0028 (10)
N2	0.0582 (17)	0.0452 (15)	0.0291 (12)	0.0205 (13)	0.0062 (12)	0.0065 (10)
C2	0.0369 (17)	0.0448 (17)	0.0254 (14)	0.0166 (14)	0.0081 (12)	0.0043 (11)
C3	0.051 (2)	0.057 (2)	0.0256 (14)	0.0178 (17)	0.0040 (14)	0.0018 (13)
C4	0.052 (2)	0.050 (2)	0.0408 (18)	0.0150 (17)	0.0032 (15)	−0.0090 (15)
C5	0.049 (2)	0.0418 (18)	0.058 (2)	0.0192 (16)	0.0097 (17)	0.0062 (15)
C6	0.051 (2)	0.055 (2)	0.0384 (17)	0.0273 (17)	0.0093 (15)	0.0133 (14)
O14W	0.0480 (13)	0.0359 (11)	0.0390 (11)	0.0149 (10)	0.0075 (10)	0.0070 (8)
O15W	0.0356 (11)	0.0438 (12)	0.0357 (10)	0.0190 (9)	0.0090 (9)	0.0048 (8)

### Geometric parameters (Å, º)

**Table:**

Cu1—O11W	2.398 (2)	N1—C2	1.347 (3)
Cu1—O11W^i^	2.398 (2)	N1—C6	1.353 (4)
Cu1—O12W^i^	1.935 (2)	N2—H2A	0.8600
Cu1—O12W	1.935 (2)	N2—H2B	0.8600
Cu1—O13W	1.9790 (18)	N2—C2	1.319 (4)
Cu1—O13W^i^	1.9790 (18)	C2—C3	1.412 (4)
O11W—H11A	0.8397	C3—H3	0.9300
O11W—H11B	0.8396	C3—C4	1.341 (4)
O12W—H12A	0.8396	C4—H4	0.9300
O12W—H12B	0.8394	C4—C5	1.397 (4)
O13W—H13A	0.8398	C5—H5	0.9300
O13W—H13B	0.8398	C5—C6	1.355 (4)
S1—O11	1.471 (2)	C6—H6	0.9300
S1—O12	1.466 (2)	O14W—H14A	0.8397
S1—O13	1.464 (2)	O14W—H14B	0.8397
S1—O14	1.462 (2)	O15W—H15A	0.8399
N1—H1	0.8600	O15W—H15B	0.8401
			
O11W^i^—Cu1—O11W	180.0	O14—S1—O11	109.47 (12)
O12W—Cu1—O11W	92.90 (9)	O14—S1—O12	109.29 (15)
O12W^i^—Cu1—O11W^i^	92.90 (9)	O14—S1—O13	109.78 (13)
O12W^i^—Cu1—O11W	87.10 (9)	C2—N1—H1	118.2
O12W—Cu1—O11W^i^	87.10 (9)	C2—N1—C6	123.6 (2)
O12W^i^—Cu1—O12W	180.0	C6—N1—H1	118.2
O12W—Cu1—O13W	89.66 (8)	H2A—N2—H2B	120.0
O12W^i^—Cu1—O13W^i^	89.66 (9)	C2—N2—H2A	120.0
O12W^i^—Cu1—O13W	90.34 (8)	C2—N2—H2B	120.0
O12W—Cu1—O13W^i^	90.34 (8)	N1—C2—C3	116.8 (3)
O13W^i^—Cu1—O11W	88.14 (8)	N2—C2—N1	120.1 (2)
O13W—Cu1—O11W^i^	88.14 (8)	N2—C2—C3	123.1 (3)
O13W^i^—Cu1—O11W^i^	91.86 (8)	C2—C3—H3	119.9
O13W—Cu1—O11W	91.86 (8)	C4—C3—C2	120.1 (3)
O13W—Cu1—O13W^i^	180.00 (11)	C4—C3—H3	119.9
Cu1—O11W—H11A	115.8	C3—C4—H4	119.3
Cu1—O11W—H11B	122.9	C3—C4—C5	121.3 (3)
H11A—O11W—H11B	104.7	C5—C4—H4	119.3
Cu1—O12W—H12A	119.8	C4—C5—H5	120.9
Cu1—O12W—H12B	125.3	C6—C5—C4	118.2 (3)
H12A—O12W—H12B	114.2	C6—C5—H5	120.9
Cu1—O13W—H13A	118.5	N1—C6—C5	119.9 (3)
Cu1—O13W—H13B	113.2	N1—C6—H6	120.0
H13A—O13W—H13B	108.7	C5—C6—H6	120.0
O12—S1—O11	108.78 (12)	H14A—O14W—H14B	106.1
O13—S1—O11	110.06 (13)	H15A—O15W—H15B	106.7
O13—S1—O12	109.43 (13)		
			
N1—C2—C3—C4	0.0 (4)	C3—C4—C5—C6	0.3 (5)
N2—C2—C3—C4	−179.7 (3)	C4—C5—C6—N1	0.0 (5)
C2—N1—C6—C5	−0.3 (5)	C6—N1—C2—N2	179.9 (3)
C2—C3—C4—C5	−0.2 (5)	C6—N1—C2—C3	0.3 (4)

Symmetry code: (i) −*x*+1, −*y*+1, −*z*+1.

### Hydrogen-bond geometry (Å, º)

**Table:**

*D*—H···*A*	*D*—H	H···*A*	*D*···*A*	*D*—H···*A*
O11*W*—H11*A*···O14*W*^ii^	0.84	2.00	2.821 (3)	167
O11*W*—H11*B*···O12^iii^	0.84	2.22	3.032 (4)	162
O12*W*—H12*A*···O15*W*	0.84	1.88	2.719 (3)	172
O12*W*—H12*B*···O13	0.84	1.85	2.677 (3)	171
O13*W*—H13*A*···O14^iv^	0.84	1.90	2.733 (3)	174
O13*W*—H13*B*···O14*W*	0.84	1.88	2.706 (3)	168
N1—H1···O13	0.86	2.03	2.855 (3)	160
N2—H2*A*···O11	0.86	2.01	2.869 (3)	176
N2—H2*B*···O12^v^	0.86	2.05	2.914 (3)	178
O14*W*—H14*A*···O15*W*^iii^	0.84	1.92	2.758 (3)	174
O14*W*—H14*B*···O14^iii^	0.84	1.90	2.738 (3)	176
O15*W*—H15*A*···O11^vi^	0.84	1.93	2.761 (3)	169
O15*W*—H15*B*···O12^iii^	0.84	1.93	2.760 (3)	170

Symmetry codes: (ii) −*x*, −*y*+1, −*z*+1; (iii) −*x*, −*y*, −*z*+1; (iv) *x*, *y*+1, *z*; (v) −*x*+1, −*y*, −*z*+2; (vi) −*x*+1, −*y*, −*z*+1.
