# Supplementary figures and images for: Crystal structure of new organically templated copper sulfate with 2-amino­pyridinium
Source: Acta Crystallogr E Crystallogr Commun. 2015 Oct 10;71(Pt 11):m191–2. doi: 10.1107/S2056989015018629 (PMC4645056; doi:10.1107/S2056989015018629)

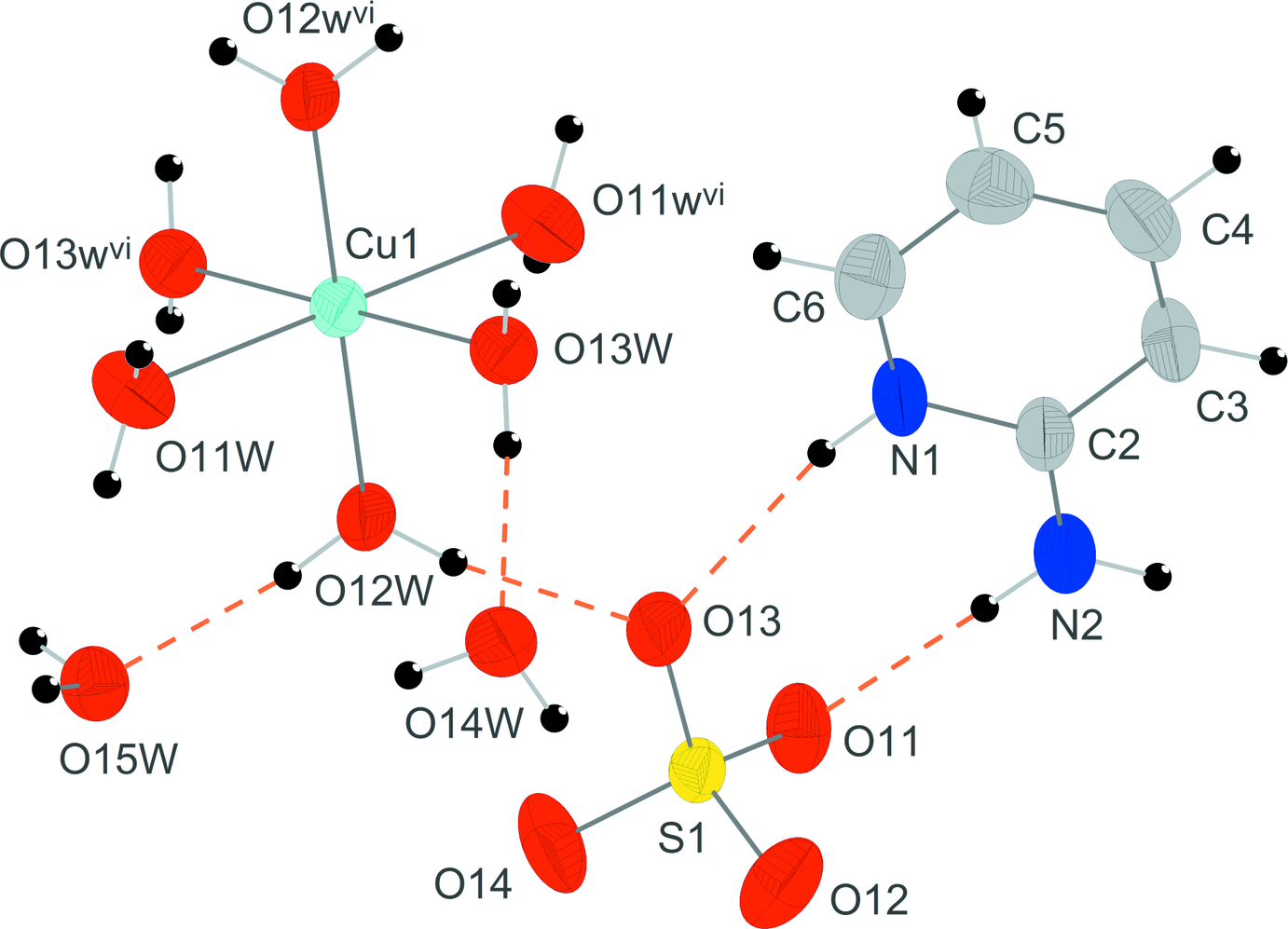

Supplement: Supplementary file 3 [file e-71-0m191-fig1.tif]

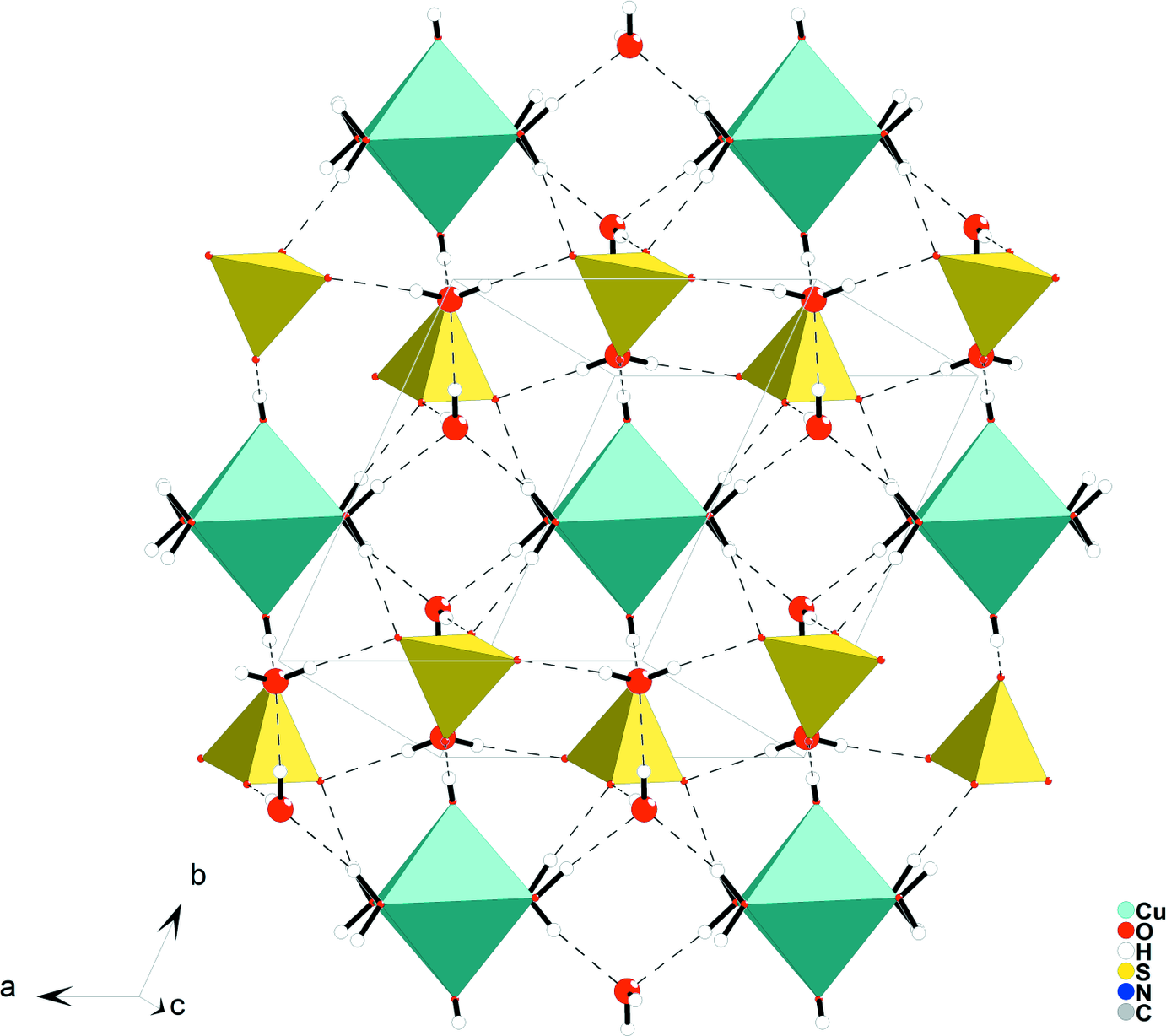

Supplement: Supplementary file 4 [file e-71-0m191-fig2.tif]

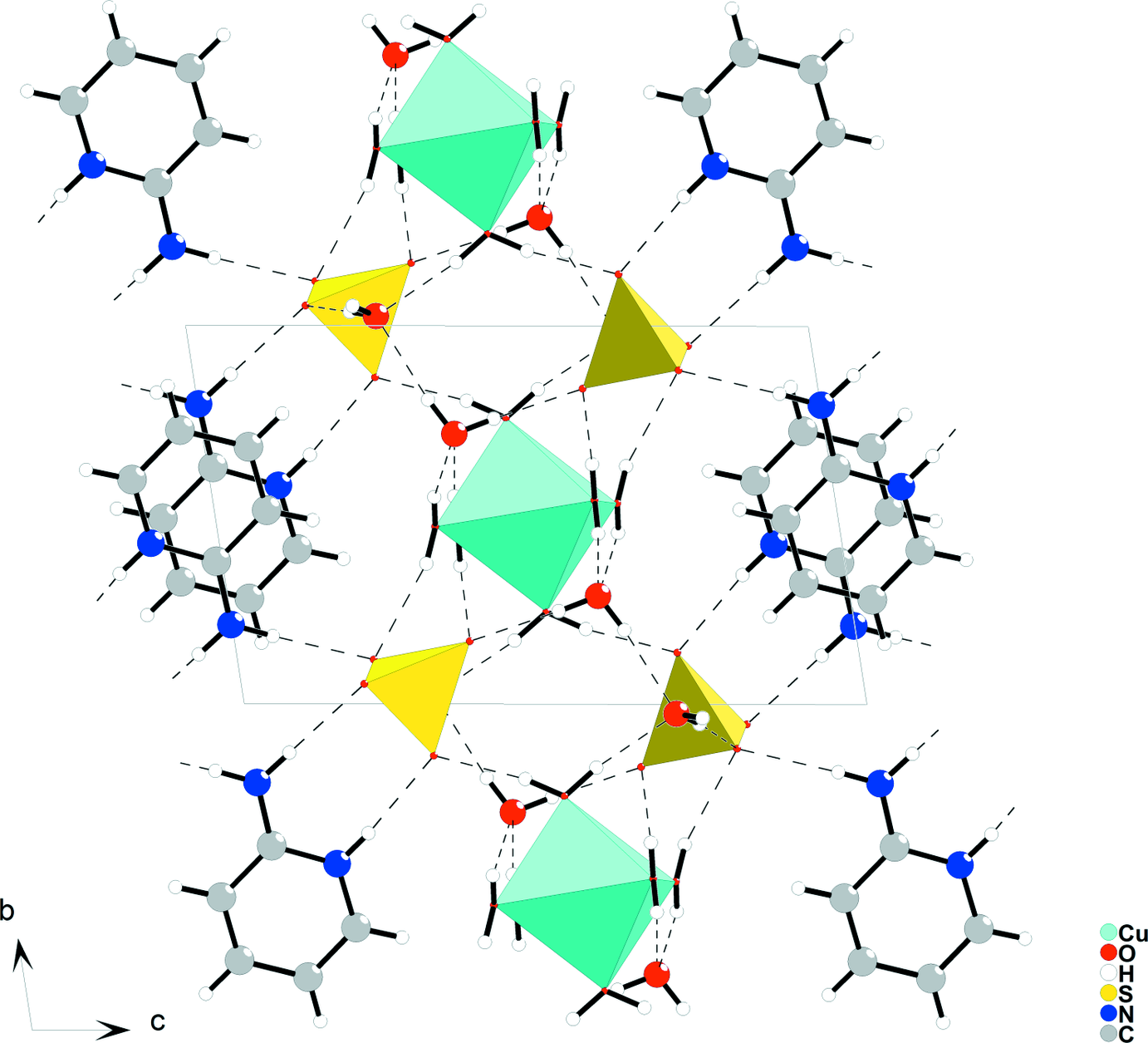

Supplement: Supplementary file 5 [file e-71-0m191-fig3.tif]
